# Performance of different colorectal cancer screening strategies: a long-term passive follow-up population-based screening program in Beijing, China

**DOI:** 10.1186/s12889-023-16564-0

**Published:** 2023-08-28

**Authors:** Xi Zhang, Lei Yang, Shuo Liu, Huichao Li, Qingyu Li, Haoxin Li, Ning Wang, Jiafu Ji

**Affiliations:** 1 Key Laboratory of Carcinogenesis and Translational Research (Ministry of Education/Beijing), Beijing Office for Cancer Prevention and Control, Peking University Cancer Hospital & Institute, Beijing, 100142 People’s Republic of China; 2 Key Laboratory of Carcinogenesis and Translational Research (Ministry of Education/Beijing), Gastrointestinal Cancer Center, Peking University Cancer Hospital and Institute, Beijing, 100142 People’s Republic of China

**Keywords:** Colorectal cancer screening, Colonoscopy compliance, Performance evaluation, Risk assessment questionnaire, Fecal immunochemical test

## Abstract

**Background:**

We aimed to assess the performance of the risk assessment questionnaire and fecal immunochemical test (FIT) in a population-based colorectal cancer (CRC) screening program to provide timely evidence for tailored screening strategies in China.

**Methods:**

This analysis was conducted using data from Beijing Cancer Screening Prospective Cohort Study (BCSPCS). A risk assessment questionnaire and FIT were selected as the primary screening methods, and participants with any positive results were referred to undergo a diagnostic colonoscopy.

**Results:**

From 2015 to 2020, 148,636 Beijing residents aged 40–69 years were invited from designated communities, with 147,807 finishing the risk assessment questionnaire and 115,606 (78.2%) completing the FIT. Among the 42,969 (29.1%) high-risk CRC participants, 23,824 (55.4%) underwent colonoscopy. One year after enrollment, all subjects were linked to the Beijing Cancer Registry (BCR) database and 241 cases of CRC were confirmed. The CRC incidence rate was 58.2/100,000 for the low-risk arm and 418.9/100,000 for the high-risk arm. For participants who underwent colonoscopy, 91 CRC cases were detected, with a detection rate of 91.9% and 63.7% of them were early-stage cases. Furthermore, the sensitivities of utilizing the risk assessment questionnaire alone, FIT alone, combined risk assessment questionnaire and FIT were 75.7%, 50.1%, and 95.1%, and the specificities were 75.3%, 87.3%, and 70.7%, respectively.

**Conclusion:**

The Beijing CRC screening program can effectively detect early-onset CRC; however, the compliance with colonoscopy still needs to be improved.

**Supplementary Information:**

The online version contains supplementary material available at 10.1186/s12889-023-16564-0.

## Background

Colorectal cancer (CRC) is the third most commonly diagnosed cancer worldwide, with an estimated 1.9 million new diagnoses, and 935,000 deaths are expected to occur in 2020 [1]. In China, there were an estimated 408,000 new cases diagnosed with CRC in 2016, accounting for 10.0% of all newly diagnosed cancer cases [2]. Urbanization, an aging population, a sedentary lifestyle, and a shift toward a Westernized diet have led to an increasing disease burden of CRC in China. As a result, CRC incidence and mortality rates in China have steadily increased over the past three decades, with the age-standardized incidence rate increasing by 2.3% annually from 1990 to 2016 [3]. Of more concern, due to health resource constraints and the lack of a comprehensive national CRC screening program, more than half (51.4%) of Chinese CRC patients had progressed to an advanced stage by the time of initial diagnosis, which dramatically reduces their survival [4].

Studies from developed countries have highlighted the long-term benefits of early detection of cancer and removal of precancerous polyps in asymptomatic individuals [5–7]. However, in developing countries, where the burden of CRC mortality has been growing sharply in recent decades, the comprehensive implementation of the CRC screening program has not yet been widely adopted [8]. Therefore, a large-scale CRC screening program with good accuracy and access to confirmatory diagnosis and treatment is urgently needed in developing countries.

Many studies have proven that stool-based tests for blood are potential methods to improve CRC detection rates since they are non-invasive, simple to administer, and cost-effective [9–12]. Moreover, risk assessment questionnaires are frequently employed to narrow the participation pool for colonoscopy, especially when a program is undertaken in a population that has never been previously screened for CRC [13–16]. Fecal immunochemical test (FIT) and risk assessment questionnaires were used as tools for initial screening for CRC in many countries, however, these studies lacked strict quality control or had no long-term health outcomes. Therefore, it is critical to assess the practical performance of various screening strategies in real-world settings.

Beijing, China’s capital city, launched an organized community-based cancer screening program (Beijing Cancer Screening Prospective Cohort Study, BCSPCS) to screen CRC and other common cancers. In the present study, we reported CRC screening results in Beijing between 2015 and 2020. The objectives of this study were to assess the real-world performance of the risk assessment questionnaire and FIT for primary CRC screening. We also evaluated the uptake of colonoscopy and factors associated with colonoscopy participation in a central metropolitan area of China. We hypothesized that our findings might yield recommendations to update screening guidelines and develop tailored strategies for CRC screening in China and other low- and middle-income countries.

## Methods

### Study design and population

This study was a community-based prospective CRC screening cohort conducted in nine district (Chaoyang, Fengtai, Shunyi, Fangshan, Huairou, Tongzhou, Daxing, Mentougou, Pinggu) of Beijing from January 2015 to December 2020. One hundred and twenty community health service centers participated in population recruitment and risk questionnaire assessment. Thirty-one officially designated tertiary-level hospital were responsible for colonoscopy examination. Generally, residents aged 40 to 69 years living in the selected communities were primarily recruited through personal encounters or telephone calls by trained primary health providers. Community advertising and social media were utilized to raise public awareness of the CRC screening program. The screening contains two steps: initial screening, inclusive of both risk assessment questionnaire and FIT, followed by diagnostic testing of a free colonoscopy for individuals with positive results of either risk assessment questionnaire or FIT. This study was approved by the Clinical Research Ethics Committee of Peking University Cancer Hospital (Approval number: 2020YJZ65). Written informed consent was obtained from each participant prior to implementation.

**Fig. 1:**
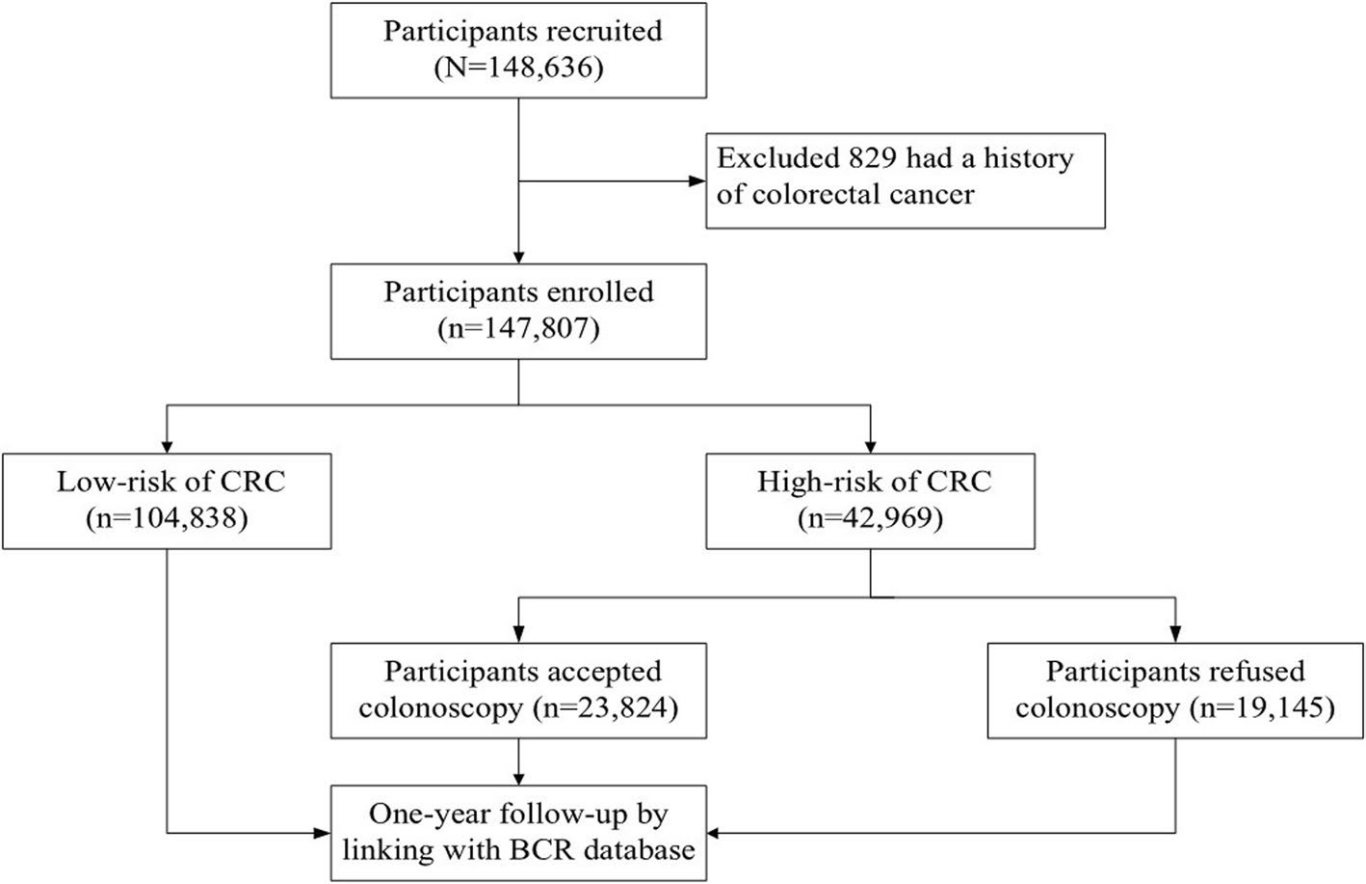
Study flowchart of participant’s enrollment, screening and follow-up of colorectal cancer screening in BCSPCS, 2015–2020. BCR, Beijing Cancer Registry; BCSPCS: Beijing Cancer Screening Prospective Cohort Study; CRC, colorectal cancer

A total of 148,636 individuals from the designated communities were recruited for this CRC screening program. After excluding 829 individuals with a prior diagnosis of colorectal cancer at baseline, 147,807 remaining participants were enrolled in the present analysis. The study flowchart is shown in Fig. 1.

### Risk assessment questionnaire

Face-to-face interviews were performed by trained healthcare staff for all participants using a paper-based questionnaire to acquire demographic information and potential CRC risk factors. Specifically, personal characteristics, including the personal identification number, age, gender, marital status, education level, smoking, and alcohol consumption, were collected through self-report. Height and weight were measured, and body mass index (BMI) was calculated as weight in kilograms divided by height in meters squared. We used an established CRC risk assessment system recommended by the Chinese consensus of early CRC screening [17]. In brief, individuals meeting one of the following criteria were identified as being at a high-risk for CRC: (i) having a personal history of colonic polyps; (ii) having a family history of CRC in first-degree relatives; or (iii) presenting with at least two of the following symptoms or signs: mucous blood stool, major mental trauma or painful event, chronic constipation, diarrhea, appendicitis or biliary disease, history of appendectomy or cholecystectomy.

### FIT procedure

The healthcare staff offered each participant a FIT kit (W.H.P.M., Inc. Beijing, China) and explained how to collect the fecal samples. Each participant collected fecal samples at home and was asked to return the samples to the healthcare center within 48 h after collection. The test result was considered positive when the sample contained a haemoglobin concentration of ≥ 100 ng/ml, which corresponds to ≥ 20 μg Hb/g faeces.

### Colonoscopy procedure

The procedures for colonoscopy were consistent with routine clinical practices in this study, including obtaining informed consent for colonoscopy, and bowel preparation. Colonoscopies were performed at the tertiary-level hospital by gastroenterologists with at least five years of experience in endoscopy. Abnormal findings during colonoscopy were carefully checked and photographed in accordance with standard clinical procedures, and biopsy samples were collected for further pathological diagnoses if necessary. Clinical information on tumor features were collected and recorded in a data system. All images for colonoscopy were stored and transferred to the research center.

### Data management and quality control

Paper-based risk assessment questionnaire, colonoscopy form, and pathology report were filled out by trained healthcare staff and physicians. The healthcare staff or physicians double-checked the data to ensure that there were no missing values or obvious logical errors. Data from the paper documents were then entered into the internet-based data management system by entry clerks at each healthcare facility. After completing the data entry, researchers downloaded all the original data, performed logical mistake verification of the data quality, and performed further analyses.

### Follow-up data

One year after the high-risk assessment, we linked all participants' identification numbers with the Beijing Cancer Registry (BCR) database to track their outcomes (diagnosed CRC or not) [18]. BCR was a population-based cancer registry covering 13 million (nearly 100%) permanent residents in Beijing [19]. The data of BCR has high accuracy and has been accepted by the International Association of Cancer Registries (IACR) as content for Cancer Incidence in Five Continents (CI5) vol. XI [20].

CRC cases were classified by site according to the *International Statistical Classification of Disease and Related Health Problems Tenth Revision (ICD-10)*. Staging of CRC was performed according to the 8^th^ edition of the American Joint Committee on Cancer (AJCC) tumor-node-metastasis (TNM) staging system [21].

### Statistical analysis

All statistical analyses were performed utilizing SAS software, version 9.4 (SAS Institute Inc., Cary, NC, USA). The socio-demographic characteristics of the participants were described by the mean and standard deviation (SD) of continuous variables or the proportion and percentage of categorical variables. *Chi*-square test was employed for comparison differences in participation rates and detection rates between groups. Univariate and multivariate logistic regressions were employed to analyze predictor variables associated with colonoscopy acceptability. The parameters that were found to be significant (*p* < 0.10) by univariate analysis were incorporated and examined using multivariate analysis, and only those variables with a *p* < 0.05 were retained in the final multivariate model. Odds ratios (ORs) and adjusted ORs with corresponding 95% confidence intervals (CIs) were calculated and reported using Wald *chi*-square statistics. Sensitivity and specificity were calculated and compared with McNemar’s test, using the data from residents with both risk assessment questionnaire and FIT results. The area under the curve (AUC) of the receiver operating characteristics (ROC) and their 95% CIs of different screening strategies were evaluated and compared using the *Z* test. All tests were two-tailed tests with a significance level of 0.05.

## Results

### Participant characteristics

Table 1 illustrates the characteristics of the participants in this study. Overall, more women (60.1%) were included in this study. The mean age was 57.6 ± 7.3 years, with 82.4% of the participants aged 50–69 years old. Approximately 58.1% of the participants were overweight or obese, and most of them had no history of bowel inflammation (95.3%), colonic polyps (96.7%) and no family history of CRC in the first-degree relative (97.8%).

**Table 1:** Characteristics of the study population among different screening measures, n (%)

Characteristics	Total	Risk assessment positive	FIT	FIT positive	At high-risk of CRC
Overall	147,807 (100.0)	37,040 (25.1)	115,606 (78.2)	15,053 (13.0)	42,969 (29.1)
Gender					
Male	59,025 (39.9)	13,304 (22.5)	46,127 (39.9)	5,682 (12.3)	15,542 (26.3)
Female	88,782 (60.1)	23,736 (26.7)	69,479 (60.1)	9,371 (13.5)	27,427 (30.9)
Age, years					
40–49	26,036 (17.6)	5,891 (22.6)	20,738 (17.9)	2,606 (12.6)	6,922 (26.6)
50–59	59,942 (40.6)	16,240 (27.1)	47,524 (41.1)	6,482 (13.6)	18,784 (31.3)
60–69	61,829 (41.8)	14,909 (24.1)	47,344 (41.0)	5,965 (12.6)	17,263 (27.9)
BMI, kg/m^2a^					
< 18.5	7,663 (5.3)	2,368 (30.9)	6,071 (5.3)	886 (14.6)	2,691 (35.1)
18.5–23.9	53,425 (36.7)	11,831 (22.1)	41,782 (36.5)	4,758 (11.4)	13,853 (25.9)
24.0–27.9	68,208 (46.8)	17,099 (25.1)	53,625 (46.9)	6,974 (13.0)	19,903 (29.2)
≥ 28.0	16,425 (11.3)	5,005 (30.5)	12,862 (11.2)	1,863 (14.5)	5,666 (34.5)
Education, years^a^					
≤ 9	32,472 (22.3)	6,721 (20.7)	25,950 (22.7)	2,616 (10.1)	7,742 (23.8)
10–12	95,078 (65.2)	24,363 (25.6)	75,369 (65.8)	9,970 (13.2)	28,220 (29.7)
≥ 13	18,275 (12.5)	5,364 (29.4)	13,226 (11.5)	2,075 (15.7)	6,277 (34.3)
History of bowel inflammation					
No	140,837 (95.3)	32,601 (23.1)	110,120 (95.3)	13,387 (12.2)	38,198 (27.1)
Yes	6,970 (4.7)	4,439 (63.7)	5,486 (4.7)	1,666 (30.4)	4,771 (68.5)
History of colonic polyps					
No	142,925 (96.7)	32,158 (22.5)	111,945 (96.8)	13,815 (12.3)	38,087 (26.6)
Yes	4,882 (3.3)	4,882 (100.0)	3,661 (3.2)	1,238 (33.8)	4,882 (100.0)
Family history of CRC in a first-degree					
No	144,524 (97.8)	33,757 (23.4)	112,987 (97.7)	14,374 (12.7)	39,686 (27.5)
Yes	3,283 (2.2)	3,283 (100.0)	2,619 (2.3)	679 (25.9)	3,283 (100.0)

*BMI* Body mass index (calculated as weight (kg)/height (m)^2^), *CRC* Colorectal cancer, *FIT* Fecal immunochemical test

^a^The total number varies due to the missing values

### Risk assessments

All participants finished the risk assessment questionnaire, of them 25.1% were assessed as high-risk of CRC by questionnaire. Furthermore, 115,606 (78.2%) participants completed the FIT and 13.0% had a positive result. As a result, 42,969 (29.1%) participants were identified as having a high risk of CRC (positive on either the risk assessment questionnaire or FIT). Females (30.9%), those aged 50–59 years (31.3%), and those who were received higher education (more than 12 years) (34.3%) were more likely to be judged as high-risk for CRC (Table 1).

### Colonoscopy participation rates and factors affecting colonoscopy compliance

Of the 42,969 CRC high-risk individuals, 23,824 underwent colonoscopy examination, yielding a participation rate of 55.4% in this study. We performed a logistic regression analysis of factors that might affect decision-making regarding colonoscopy compliance for CRC high-risk individuals. The findings revealed that participants who were aged 40–49 years, and who received higher education had relatively higher colonoscopy compliance than other groups (*p* < 0.05). Furthermore, participants who had a history of bowel inflammation, colonic polyps, or had a family history of CRC in their first-degree were more likely to undertake colonoscopy. Additionally, individuals who were assessment as high-risk of CRC by questionnaires were more likely to undergo colonoscopy than those with positive FIT results (*p* < 0.001). Gender and BMI had no significant effect on decision-making regarding colonoscopies (*p* > 0.05) (Table 2).

**Table 2:** Factors associated with colonoscopy compliance for CRC high-risk individuals in Beijing

Factors	Not underwent colonoscopy, n (%)	Underwent colonoscopy, n (%)	*OR* (95% *CI*)	*p* value	Adjusted *OR*^a^ (95% *CI*)	*p* value
Gender						
Males	6,883 (44.3)	8,659 (55.7)	1.00			
Females	12,262 (44.7)	15,165 (55.3)	0.98 (0.95–1.02)	0.399		
Age, years						
40–49	2,894 (41.8)	4,028 (58.2)	1.00		1.00	
50–59	8,072 (43.0)	10,712 (57.0)	0.95 (0.90–1.01)	0.094	0.97 (0.92–1.03)	0.296
60–69	8,179 (47.4)	9,084 (52.6)	0.80 (0.75–0.84)	< 0.001	0.83 (0.78–0.88)	< 0.001
BMI, kg/m^2^						
< 18.5	1,182 (43.9)	1,509 (56.1)	1.00			
18.5–23.9	6,246 (45.1)	7,607 (54.9)	0.95 (0.88–1.04)	0.267		
24.0–27.9	8,780 (44.1)	11,123 (55.9)	0.99 (0.92–1.08)	0.852		
≥ 28.0	2,506 (44.2)	3,160 (55.8)	0.99 (0.90–1.08)	0.793		
Education, years						
≤ 9	3,862 (49.9)	3,880 (50.1)	1.00		1.00	
10–12	12,404 (44.0)	15,816 (56.0)	1.27 (1.21–1.34)	< 0.001	1.20 (1.14–1.27)	< 0.001
≥ 13	2,519 (40.1)	3,758 (59.9)	1.49 (1.39–1.59)	< 0.001	1.37 (1.28–1.47)	< 0.001
History of intestinal inflammation						
No	17,205 (45.0)	20,993 (55.0)	1.00		1.00	
Yes	1,940 (40.7)	2,831 (59.3)	1.20 (1.13–1.27)	< 0.001	1.17 (1.10–1.24)	< 0.001
History of colonic polyps						
No	17,238 (45.3)	20,849 (54.7)	1.00		1.00	
Yes	1,907 (39.1)	2,975 (60.9)	1.29 (1.21–1.37)	< 0.001	1.28 (1.20–1.36)	< 0.001
Family history of CRC in a first-degree						
No	17,821 (44.9)	21,865 (55.1)	1.00		1.00	
Yes	1,324 (40.3)	1,959 (59.7)	1.21 (1.12–1.30)	< 0.001	1.17 (1.08–1.26)	< 0.001
Initial screening methods						
FIT	2,730 (46.0)	3,199 (54.0)	1.00		1.00	
Questionnaire	12,447 (44.6)	15,469 (55.4)	1.06 (1.00–1.12)	0.040	1.02 (0.97–1.09)	0.430
Both	3,968 (43.5)	5,156 (56.5)	1.11 (1.04–1.18)	0.002	1.09 (1.02–1.17)	0.013

*BMI* Body mass index (calculated as weight (kg)/height (m)^2^), *CI* Confidence interval, *CRC* Colorectal cancer, *FIT* Fecal immunochemical test, *OR* Odds ratio

^a^Odds ratios were adjusted for factors including gender, age, marriage status, body mass index, education background, screening methods in the logistic regression models

### Detection rates of colorectal lesions in different age groups and gender

As shown in Table 3, the detection rates for CRC, advanced adenomas, and non-advanced adenomas by colonoscopy increased with age (*p* < 0.001). Compared with females, males consistently showed higher detection rates for CRC, advanced adenomas, and non-advanced adenomas by colonoscopy (*p* < 0.01).

**Table 3:** Detection of colorectal lesions in different age groups using colonoscopy

Characteristics	Colonoscopies	CRC, n (%)	Advanced adenomas, n (%)	Non-advanced adenomas, n (%)
Age, years				
40–49	4,028	3 (0.1)	94 (2.3)	395 (9.8)
50–59	10,712	25 (0.2)	377 (3.5)	1,528 (14.3)
60–69	9,084	56 (0.6)	465 (5.1)	1,630 (17.9)
*p*		< 0.001	< 0.001	< 0.001
Gender				
Males	8,659	44 (0.5)	510 (5.9)	1,743 (20.1)
Females	15,165	40 (0.3)	425 (2.8)	1,810 (11.9)
*p*		0.002	< 0.001	< 0.001
Total	23,824	84 (0.4)	935 (3.9)	3,553 (14.9)

*CRC* Colorectal cancer

### CRC detected in the screening and non-participant screening groups

After one year of passive follow-up, 241 CRC cases were confirmed by matching to the BCR database, with an overall incidence rate of 163.1/100,000 (241/147,807). The incidence rate of CRC was 58.2/100,000 (61/104,838) in the low-risk group and 418.9/100,000 (180/42,969) in the high-risk group. Among the low-risk participants, 61 cases were matched with the BCR database, of which early-stage patients accounted for 38.2% (13/34). For participants who underwent colonoscopy, 136 CRC patients were diagnosed, including 69 colon cancers and 67 rectal cancers. In addition, 91 CRC patients were detected, with a detection rate of 91.9% (91/99) and 63.7% (58/91) were early-stage cases. However, 22 CRC patients were missed to be diagnosed, with a missed diagnosis rate of 8.1% (8/99) and 50.0% (4/8) being early cases. Additionally, for high-risk participants who did not complete colonoscopy, 44 CRC patients (16 in the colon and 18 in the rectum) were matched with the BCR database, of which 48.0% (12/25) were early-stage cases (Table 4).

**Table 4:** Characteristics of detected and undetected CRC

Characteristics	Low-risk of CRC (*n* = 104,838)	High-risk of CRC (*n* = 42,969)			
		**Non-participant screening (*n* = 19,145)**	**Screening (*n* = 23,824)**		
			**Screened**	**Detected**	**Undetected**
Tumor location					
Colon	33	16	69	58	11
Rectum	28	18	67	56	11
Total	61	44	136	114	22
Incidence rate	58.2/100,000	229.8/100,000	570.9/100,000	478.5/100,000	92.3/100,000
Cases with TNM stage	34	25	99	91	8
Early-stage cases (rates)	13 (38.2)	12 (48.0)	62 (62.6)	58 (63.7)	4 (50.0)

*CRC* Colorectal cancer, *TNM* Tumor, node, metastasis

### Performance of different CRC screening strategies

After data linkage with the BCR database, we compared the performance of three different CRC primary screening strategies in this study, including risk assessment questionnaire alone (model 1), FIT alone (model 2), and risk assessment questionnaire and FIT co-testing (model 3). As presented in Table 5, the sensitivities of the preceding three strategies were 75.7%, 50.1%, and 95.1%, while the specificities were 75.3%, 87.3%, and 70.7%, respectively. McNemar’s test showed significant differences in the sensitivity and specificity between these three groups (all *p* < 0.001). Moreover, model 3 had the highest AUC (0.829), followed by model 1 (0.755) and model 2 (0.687), with statistically significant differences across the three groups (all *p* < 0.001).

**Table 5:** Comparison of performance of different CRC screening strategies^*^

Methods	Advanced adenoma + CRC		
	**model 1**	**model 2**	**model 3**
Sensitivity, % (95%CI)	75.7 (73.1–78.1)	50.1 (46.8–53.3)	95.1 (93.5–96.3)
Specificity, % (95%CI)	75.3 (75.1–75.6)	87.3 (87.1–87.5)	70.7 (70.5–71.0)
AUC, (95%CI)	0.755 (0.741–0.769)	0.687 (0.667–0.707)	0.829 (0.820–0.839)

*AUC* The area under the receiver operating characteristic curve, *CI* Confidence interval, *CRC* Colorectal cancer, *FIT* Fecal immunochemical test, *model 1* Risk assessment questionnaire alone, *model 2* FIT alone, *model 3* Risk assessment questionnaire and FIT co-testing

^*^Only residents with risk assessment questionnaire and FIT results were included

## Discussion

This study presents the results of 147,807 individuals who participated in the CRC screening program in Beijing during 2015 to 2020. We assessed colonoscopy compliance in high-risk citizens and explored its associated influencing factors. In addition, the long-term passive follow-up confirmed the participants' health outcomes to evaluate the detection rates of CRC screening, as well as reported diagnostic yield for multiple screening strategies. These results highlight the importance of a diversity of screening strategies and provide evidence to promote future improvements in CRC screening effectiveness. Furthermore, these real-world practice data have important policy implications and generalizability to other developing countries.

Colonoscopy has been the dominant method for CRC screening in many countries. For instance, the U.S. Preventive Services Task Force (USPSTF) recommended colonoscopy as the screening method for CRC in asymptomatic adults aged 50 to 75 years at average risk [22]. Although there is sufficient scientific evidence to support that colonoscopy reduces CRC mortality [23–25], the acceptance of colonoscopy is still suboptimal in many countries, especially when colonoscopy is used as the primary screening modality. A population-based randomized clinical trial (RCT) conducted in four European countries (Poland, Norway, the Netherlands, and Sweden) found that the participation rates of colonoscopy varied significantly across countries, from 22.9% to 64.7% [26]. Screening programs conducted in different regions in China showed a similar scenario. Specifically, colonoscopy participation rates were reported to range from 14.0% to 39.8% among people at high risk of CRC in China [27–29]. Therefore, poor compliance with colonoscopy is a common issue worldwide. In our analysis, the participation rate of colonoscopy among the high-risk population of CRC was 55.4%, similar to that in Shanghai [30], but far away from that in the US (65%) [31].

We attribute the low colonoscopy compliance in this program to three main reasons. First, due to a lack of awareness regarding screening, several residents mistakenly believed that colonoscopy was only necessary if they had symptoms such as blood in the stool or lower abdominal pain [32, 33]. As a result, despite having a positive result from a risk assessment questionnaire or FIT, they refused to undergo colonoscopy. Previous studies suggested that participants’ awareness and knowledge about CRC screening was an important factor for a successful CRC screening program [33–35]. To overcome these barriers, considerable effort should be made to develop educational and outreach programs to improve compliance of high-risk populations. Second, because our study used a painful colonoscopy, some residents refused to undergo a colonoscopy because they were afraid of pain and discomfort associated with the test [36, 37]. This demonstrates that offering a painless colonoscopy option for CRC screening might significantly increase compliance and screening participation. Recently, computed tomographic (CT) colonoscopy, an imaging method based on scanning technology, has been developed as a less invasive visualization technique for CRC screening [38]. These methods may provide a new option for people who are reluctant to undergo colonoscopy due to fear of pain. Third, colonoscopy requires approximately one week of preparatory time for infectious disease screening and bowel preparation, and some residents have declined to undergo colonoscopy because of inconvenience [39]. Thus, in future CRC screening, the process of screening should be optimized and unnecessary procedures should be reduced.

Our study identified that younger and more educated people in the high-risk group were more motivated to undergo colonoscopy, probably because they were more concerned about their health and had more knowledge about CRC screening. In addition, we found that males were more compliant with colonoscopy than females, which was probably attributable to the painful colonoscopy employed in this study, and females were generally less likely to tolerate the discomfort of colonoscopy. The associations of these factors have been extensively explored, and our findings were in line with previous studies [32, 40]. In addition, an interesting finding was that the questionnaire-positive group had considerably higher colonoscopy attendance than the FIT-positive group. This could be because many questionnaire-positive individuals have the gastrointestinal disease themselves or have a first-degree relative with CRC. Therefore, they were more concerned about themselves, prompting them to accept a colonoscopy.

It deserves to be noted that the overall detection rate for CRC was considerably higher in the screening group than in the non-screening group. Meanwhile, the screening group had far more early cases than the non-screening group, indicating that the CRC screening program in Beijing was effective and could detect more early-onset cases than the non-screening group. In addition, the risk assessment questionnaire used in our study showed good discriminatory power (AUC = 0.755), which was similar to a risk stratification-based screening model in Korea (AUC = 0.681) [15], indicating that the risk assessment questionnaire could be applied to identify high-risk asymptomatic subjects for advanced adenomas. FIT is the most widely used non-invasive CRC screening method. However, FIT in population-based CRC screening is uncertain due to a lack of evidence. The current study found that using FIT as the primary screening tool could concentrate high-risk populations and avoid unnecessary colonoscopies. Given the relatively poor participation rate in screening, numerous CRC cases were missed during the program, which substantially reduced the effectiveness of the screening. To improve the diagnostic yield of CRC screening in Beijing, the following challenges should be further addressed. To begin, we will develop appropriate CRC screening strategies based on the current research findings for diverse demographics and different risks of CRC. Furthermore, multi-factor interventions targeting various populations should be implemented to boost colonoscopy compliance.

This study has several limitations. Firstly, selection bias may exist in this large-scale screening program. People who had symptoms (blooding fetus, chronic intestinal discomfort, etc.) or had a first-degree relative with CRC were more willing to participate in this screening program and accept colonoscopy, leading to higher positive rates of risk assessment questionnaires and FITs and compliance with colonoscopies. Secondly, we assessed CRC risk using a self-reported questionnaire. As a result, some citizens may lie to receive a free colonoscopy. Thirdly, this study did not manage individuals who had previously undergone colonoscopy testing, which may affect colonoscopy compliance and detection rates. Fourthly, Beijing boasts the top-notch healthcare facilities equipped with cutting-edge technologies of the country. In this study, the data quality was determined mainly by the experience of the gastroenterologists and healthcare professionals who conducted the interviews. Therefore, the results of Beijing cannot be applied to other Chinese cities. Finally, we used the cancer registration data as the endpoint of this study. However, the cancer registration data were approximately half a year behind the diagnostic time at the hospital, which meant that some patients would be missed in this study. Nevertheless, despite Beijing having a comprehensive cancer registration system, a tiny number of cases may still be omitted. As a result, this study’s sensitivity may be overestimated, whereas the specificity may be underestimated. Furthermore, as the current registration system does not necessitate reporting the cancer stage, there may be inaccuracies in the staging of this study.

## Conclusion

In conclusion, this large-scale population-based CRC screening program can effectively detect early-onset CRC and advanced adenomas, although compliance with colonoscopy still needs to be improved. This study provides strong evidence for the effectiveness of population-based CRC screening for policymakers to design nationwide screening programs in the future.

### Supplementary Information

**Additional file 1.**

## Data Availability

The dataset supporting the conclusions of this article are available from the corresponding author on reasonable request.

## Abbreviations

AJCC: American Joint Committee on Cancer
AUC: Area under the curve
BCR: Beijing Cancer Registry
BCSPCS: Beijing Cancer Screening Prospective Cohort Study
BMI: Body mass index
CI: Confidence interval
CI5: Cancer Incidence in Five Continents
CRC: Colorectal cancer
FIT: Fecal immunochemical test
IACR: International Association of Cancer Registries
ICD-10: International Statistical Classification of Disease and Related Health Problems Tenth Revision
OR: Odds ratios
RCT: Randomized clinical trial
ROC: Receiver operating characteristics
SD: Standard deviation
TNM: Tumor-node-metastasis
USPSTF: U.S. Preventive Services Task Force
