# Supplementary material for: Performance of different colorectal cancer screening strategies: a long-term passive follow-up population-based screening program in Beijing, China
Source: BMC Public Health. 2023 Aug 28;23:1640. doi: 10.1186/s12889-023-16564-0 (PMC10463986; doi:10.1186/s12889-023-16564-0)
Supplement: Supplementary file 1 — Additional file 1. [file 12889_2023_16564_MOESM1_ESM.pdf]

Risk Assessment Questionnaire for Colorectal Cancer

ID No. |__|__|__|__|__|__|__|__|__|__|__|__|__|__|__|__|__|__| Survey date：|__|__|__|__|year|__|__|month|__|__|day

1.General information

1.1 Gender 1=male 2=female 1.2 Marital status：1=Single 2=Married 3=Divorced 4=Widowed 5=Others

1.3 Education：1=No schooling 2=primary school 3=High school 4=Undergraduate 5=Postgraduate or above

1.4 Occupation：1=farmer 2=Worker 3=Self-employed people 4=Professional and technical staff 5=Others

1.5 Height：|__|__|__|cm 1.6 Weight：|__|__|__|kg

2. Life-style

2.1 Personality characteristics 1= Impatient and easy to get angry 2= Oversensitive and often sulks

3= Mild and rarely angry 4= Open-minded and good at communicating with others

2.2 Working mode： 1= Sedentary office jobs 2= Work that requires walking or standing

2.3 Work stress： 1= Hard work and high pressure 2= Easy work and low pressure

2.4 Tobacco smoking 1=Yes 2=No Alcohol Drinking 1=Yes 2=No Tea drinking 1=Yes 2=No

2.5 Have you have the following eating habits? 1=Yes 2=No

2.51 Like eating red meat 2.52 Eat lots of processed meat 2.53 Like eating meat 2.54 Eating lots of vegetables

2.55 Less to eat vegetables 2.56 Exercise regularly 2.57 Lack of exercises 2.58 Drink dairy prodicts every day

3. Personal history of the following diseases 1=Yes 2=No

3.1 Ulcerative Colitis 3.2 Crohn's disease 3.3 Gastric and Duodenal Ulcer 3.4 Diabetes 3.5 Haemorrhoids

4. Family history of any cancer 1=Yes 2=No (If Yes, Who?)

5.1 Personal history of chronic diarrhea 1=Yes 2=No （Chronic diarrhea: In recent 2 years, the diarrhea lasted for more than 3 months, and each episode lasted for more than a week）

5.2 Personal history of chronic constipation? 1=Yes 2=No （Chronic constipation: In recent 2 years, constipation is more than 2 months per year）

5.3 Personal history of mucous or bloody stools 1=Yes 2=No

5.4 Personal history of appendicitis or had an appendectomy 1=Yes 2=No

5.5 Personal history of chronic cholecystitis or cholecystectomy 1=Yes 2=No

5.6 Personal history of any adverse life events in the last 20 years 1=Yes 2=No If Yes, which one?

1=Divorced 2=Death of spouse 3= Death of a first-degree relative 4=Laid off of the child 5=Others

（Adverse life events：Occurs within the last 20 years and causes significant psychological trauma or distress after the event）

5.7 Personal history of intestinal polyps 1=Yes 2=No

6. Do you have any first-degree relatives with colorectal cancer? 1=Yes 2=No 3=Unknown (If Yes, Who?)
